# Internal Structure, Invariance, and Rasch Analyses: A Work-Life Integration-Blurring Scale

**DOI:** 10.3390/healthcare10112142

**Published:** 2022-10-28

**Authors:** Juanita Hincapié Pinzón, Andressa Melina Becker da Silva, Monique Cristielle Silva da Silva, Wagner de Lara Machado, Carmen Moret-Tatay, Manoela Ziebell de Oliveira

**Affiliations:** 1 Faculty of Psychology, Universidad Católica de Valencia San Vicente Mártir, Sede Padre Jofré, Av., Ilustración nº2, 46100 Valencia, Spain; 2 Postgraduate Psychology Program, School of Health Sciences, Pontifical Catholic University of Rio Grande do Sul, Porto Alegre 90619-900, RS, Brazil; 3 Department of Psychology, University of Sorocaba, Sorocaba 18023-000, SP, Brazil; 4 Department of Physical Education, University of Sorocaba, Sorocaba 18023-000, SP, Brazil

**Keywords:** cross-cultural, blurring, psychometrics, validity, reliability

## Abstract

The aim of the study was to develop a role blurring (RB) tool to measure work-life integration in different contexts. A final number of 19 items was examined. Psychometric properties in both Spanish and Brazilian Portuguese versions were analysed, comparing the invariance of the measure between the two countries, and setting the difficulty parameter of the items. Thus, a total of two incidental samples volunteered to participate in the study: a Spanish sample of *n* = 498 and a Brazilian sample of *n* = 379 were recruited. Confirmatory factor analysis (CFA), as well as an invariance analysis, were performed between the two countries, in order to test the homogeneity structure of the measure. In addition, the item response theory (IRT) was used to establish the probability of response and difficulty of the items through a Rasch analysis. The confirmatory factor analysis pointed to a unifactorial structure. There were no statistically significant alterations in the model fit indices, and the Rasch rating scale model (RSM) showed adequate infit and outfit values, as well as successive response categories located in the expected order for all items.

## 1. Introduction

The digital era poses a challenge in balancing roles between family and work, due to the high demands that interfere and create blurring in the boundaries between work and daily life [1]. This blurring phenomenon is called role blurring (RB), and it occurs when permeated limits overlap with each other [2]. In other words, “when people are physically exercising a role, but their behaviours or thoughts are in another activities” [3].

The increase in technological interconnections may directly influence how people experience work and family life. Technology not only helps to maintain the connection, but also to monitor activities at all times. However, it may also contribute to the blurring of work and family roles that are generated by contact with work outside the working day. In this way, blurred boundaries can affect the way family and work are experienced and managed [4]. More precisely, work activities can be prolonged, generating implications for the well-being of the individual and family life as a result of the employee’s perception regarding the performance they should have, or what is expected from them [5].

By working at any time and in any place, RB could create a higher association between work-family conflict levels [6]. When work demands extend beyond the time and space allocated for work, mainly as a result of information and communication technologies (ICTs), the boundaries of other areas are crossed, generating unwanted effects [7]. From this perspective, [8] examined the physical, psychological, social, or organisational efforts during labor demands (e.g., adaptations to new routines, work pressure, emotional demands, among others). This study presupposes that there is an interaction with the resources available to meet, and their effects. Thus, it is expected that, under smart working and teleworking contexts, the boundaries between work and non-work blur [9].

Different tools were developed in this scenario. First, the work-to-life conflict, with four items, has depicted good internal consistency (α = 0.84) [10]. It is also possible to find domain flexibility with 36 items that are divided into work flexibility-ability (10 items, α = 0.84), work flexibility-willingness (9 items, α = 0.68), family flexibility-ability (9 items, α = 0.72), and family flexibility-willingness (8 items, α = 0.75) [11]. The role conflict scale has also showed good internal consistency under seven items (α = 0.86) [12], and it has been employed to measure underlying variables to RB. Moreover, work-family integration-blurring scale (WFIBS) (3 items, α = 0.73) [13], role blurring at home (5 items α = 0.65) [6], and the work–family role blurring (3 items, α = 0.65) [14], are specific for this construct under study. However, it is noteworthy that variables that underlie technologies as work aids may be missing, in terms of psychological impact in RB. Furthermore, a lack of uniqueness in the understanding of the concept suggests that the theoretical perspectives are mixed, and are even equated with correlated variables. Consequently, two dominant theories were used for the construction of the instrument in the empirical literature: the work-family border theory [15], and the border/boundary theory [3], which deal with the integration and separation of the boundaries between work and family.

*Role Blurring and COVID-19*

Since December 2019, a virus responsible for the COVID-19 pandemic created a global health crisis, with more than 511.4 million confirmed cases worldwide, including 6.2 million deaths, until 2 May 2022. In that time, more than 11.5 billion vaccinations were registered worldwide [16]. However, effective vaccines and/or drugs were not available to treat COVID-19 for more than a year. Bearing in mind that the health crisis had an impact on societies, politics, and the economy, among others [17,18], the most effective strategies to control the pandemic during this period were the preventive measures that were adopted by the governments of each country.

A comparison of populations that were affected by the sudden onset of the virus helps to understand indicators of human development, health, connectivity, and lethality during the pandemic. The impact of strategies that were taken or omitted in various areas differ in social, economic characteristics, management, and territorial extension, among others. Medina-Hernández et al. [19] pointed out differences between countries, in order to look for gap mitigation strategies; examples of this include the different working conditions in countries such as Brazil and Spain, which allow for the comparison of the resources and demands that ICT offered, thanks to teleworking, and the underlying increase in RB.

The International Work Organisation report from March 2021 described numbers of home offices of workers from 36 countries. On the one hand, Brazil had an estimated 10.7%; on the other hand, for Spain it was 16.2% [20]. Bearing in mind that the possibilities of access and connectivity to the digital world are not equitable in all countries, the impact that the use of technology as a work aid has on the boundaries between work and the family of individuals should be assessed, with appropriate measuring instruments.

For this reason, during the COVID-19 pandemic, the increase in strategies such as teleworking may have promoted the phenomenon of RB, by perceiving it more intensely and frequently as a result of its direct impact on the management of limits, not only spatial, but also temporal. Hence, the need to create an instrument to measure RB, which conceives social characteristics and changes during that period, was addressed in the current study. The aim of this paper was to develop a tool to measure RB, and to examine its psychometric properties for both Spanish and Brazilian Portuguese versions.

## 2. Materials and Methods

### 2.1. Design and Procedures

The design of the study was composed of two stages. In the first, a qualitative approach was adopted to construct and obtain evidence for the validity of the content in the proposed scale. The second stage consisted of a cross-sectional study with an incidental sampling, for the purpose of conducting a study of psychometric properties under the proposed tool. Both stages are described as follows.

Stage 1. Building and Validating Content of the Role Blurring Scale

We followed the phases of Boateng et al. [21] for the generation of items: identification of factors, and development of items.

The proposed scale was developed in three different phases. The so-called preliminary phase, pertained to the elaboration and evaluation of the contents from each item. The construction of the role blurring instrument considered the different conditions and levels of integration between the roles on the basis of two dominant theories: work-family border and border/boundary in the literature. The most relevant theoretical categories related to the phenomenon were analysed. From the search in literature, an essential category for the study emerged: RB and mental health in the digital age. In this way, an initial approach with 40 items was obtained.

Scale Development

Different levels of behaviours that were associated with the role blurring phenomenon were identified, of which four possible alternatives of variability in behavioural and psychological indicators determined the response options. The preliminary test of the questions allowed for the reduction in items to 21, and for the determination of the unifying nature of the scale. Despite the possible manifestations of role blurring that were associated with specific aspects, such as deadlines (time), the workplace (space), the relationship with technology (flexibility), and psychological manifestations (stress), the confluence in the interference mechanism by superimposition and blurring of the boundaries in the roles played by a person were considered the same. Thus, a single factor was proposed.

Evidence of Validity of Role Blurring Scale Content; Expert Assessment

In the third phase, related to content validity, a panel of experts was selected to assess the scale. Three expert judges, professionals who graduated in psychology with a PhD in the area, evaluated whether the grammatical, semantic, and idiomatic constructions of the proposed items adequately measured the construct of interest. Experts were asked to assess items 1 to 5 with respect to clarity of language, relevance, and practical relevance of items. As the variation in the level of concordance of the evaluators was low, the Finn coefficient was used as an index of reliability among evaluators of quantitative data [22]. The Finn coefficient was calculated as an index of reliability among quantitative data evaluators. The resulting instrument was used in Stage 2.

Stage 2. Cross-Sectional Study (Applied for the Following Sections)

### 2.2. Participants

A sample of 877 adults volunteered to participate in the study, 498 adults from Spain and 379 from Brazil. The age range of the Spanish participants was from 18 to 61 years, mean = 24.88 and SD = 9.68 years, 76.2% (*n* = 363) women, and 23.7% (*n* = 113) men. For the Brazilian sample, participants were between 18 and 68 years old, with 75.2% (*n* = 271) women and 24.7% (*n* = 89) men, mean = 34.5, and SD = 9.51 years.

### 2.3. Instruments

A sociodemographic list of questions was employed. It involved questions related to sex, age, among others, as well as those related to the use of technologies for work, and their impacts on the lives of the participants. Moreover, a Spanish or Portuguese version of the RB tool under 21 items with Likert response format was employed. Data collection was carried out in both countries during the months of August to December 2021.

### 2.4. Data Analysis

The analyses were performed with R Studio software (R Language for Statistical Computing) (R Core Team, 2020) and the Winsteps program [23]. The following sections describe the analysis procedures in detail. In the first stage, the calculation of the Finn coefficient was carried out, which was especially useful for the high agreement between evaluators. The random selection of evaluators from a larger group of people with the “two way” model was specified. A confirmatory factor analysis (AFC) was performed, and the test of configural, metric, scalar and strict invariance between the two countries was performed in order to test the factorial homogeneity structure between the groups using the weighted least squares diagonal extraction method (DWLS) [24]. In addition, item response theory (TRI) was used to establish the probability of response and difficulty of items, through the Rasch analysis.

R Studio was employed, particularly, IRR [25], extended Rasch modeling: The eRm package for the application of IRT models in R [26], psych [27], and qgraph [28]. A convenient way to perform multi-group confirmatory factor analyses (MG-CFA), even when the data are categorical, is the use of lavaan packages [29] and semTools [30]. The Winsteps program was used to generate the probability curve, and the differential performance chart of items.

### 2.5. Reliability

A descriptive analysis (mean and standard deviation) of the information collected was carried out. In the evaluation of the internal consistency of the instrument, the values of Cronbach’s Alpha coefficients (α) > 0.7 were satisfactory [31], as was McDonald’s Omega [32]. The confirmatory factor analysis (AFC) showed satisfactory indices, with a comparative adjustment index (CFI) and Tucker–Lewis index (TLI) > 0.9, with mean quadratic approximation error (RMSEA), and standardized mean quadratic residue (SRMR) < 0.08 [33].

A series of multi-group CFA models were adjusted, in order to check the measurement invariance of the RB instrument. We selected two independent samples of adults that were collected in Spain and Brazil. The two groups were randomly divided into the two populations. The logic for this strategy consisted of testing the factorial homogeneity structure between independent groups for invariance analysis in the development of a psychometric test [34]. The assessment of the model fit for each population (country) identifies a base model and tests the residues, followed by the invariance load with more restrictive models (i.e., thresholds and/or loads are restricted to be equal in both countries). This hierarchical procedure begins with an unrestricted model, and more constraints are added successively.

Therefore, four models of invariance were examined: configural, metric, scalar, and strict invariance. Chen [35] suggested that changes in CFI (ΔCFI) equal to or greater than 0.01, in addition of a change in RMSEA (ΔRMSEA) less than or equal to 0.015, were indicative of non-invariance. This case was proposed in single-agent models, when sample sizes were equal in all groups, with more than 300 in each group.

The items were treated as categorically ordered, as the assumption that the distances between the response options was considered accurate. In this way, the residues were estimated instead of intercepted. Specifically, the categorical logit model ordered with theta parameterisation [36] was used, together with the weighted least squares estimator (mean and adjusted variance [WLSMV]) [37].

The item response theory (TRI) method evaluates the quality of each item on the scale. The Rasch model was used to measure the reliability of items through their statistics, the probability of response categories, and the difficulty of the item in relation to the latent stroke of the person [38]. The rating scale model was estimated to evaluate, independently, the parameter of difficulty of the items [39]. An item’s fit was evaluated from the infit and outfit indices, with an expected value of 1.0. Values between 0.5 and 1.5 indicate the adherence of the responses of an item to the expected responses of the model [40].

**Figure 1:**
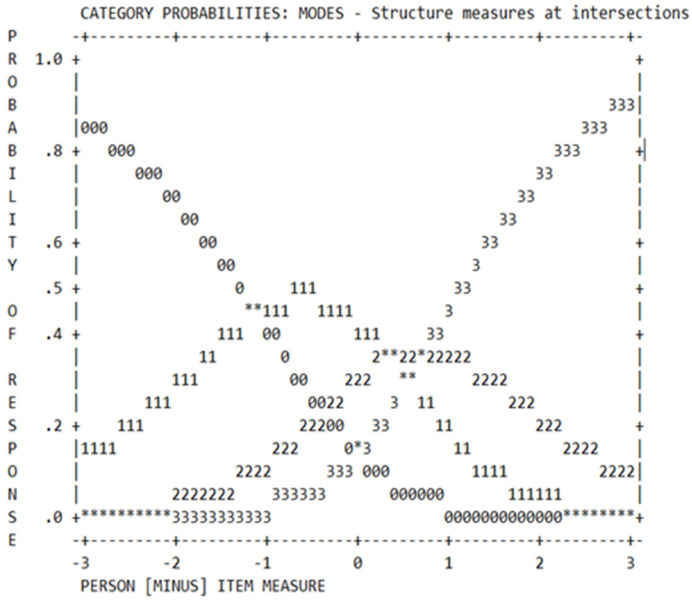
Response Probability Curve of the Role Blurring Scale. The horizontal axis represents the ability levels of respondents, while the vertical axis represents the probability of endorsement for each category. Numbers represented each category of instruments’ Likert type scale.

Subsequently, the performance of the rating scale was evaluated through scale category curves, using a probability curve analysis. In order to evaluate the function of a valuation scale (Figure 1), the probability of selecting a particular response category was examined. Finally, Boone et al. [41] explained the differential functioning of items (DIF) as the measure that considers whether the way items define a scale does the same for different groups.

### 2.6. Ethical Procedures

This study was approved by the Research Ethics Committee (Code Number: UCV/2020-2021/129 at the Catholic University of Valencia San Vicente Mártir, and Number 4.486.239 at the Pontifical Catholic University of Rio Grande do Sul). In order to comply with the ethical aspects of the research, informed consent was provided, following the resolutions of the European Union and Brazil, as well as the recommendations of Helsinki.

## 3. Results

In the descriptive analysis of the entire sample, the subsample of the Spanish population was 43.2% (*n* = 372), and the Brazilian population was 56.7% (*n* = 498). An age range between 18 and 68 years was determined for the population, with a standard deviation of 10.73. Regarding gender, 75.29% (*n* = 634) of the sample were registered as women at birth, and 23.03% (*n* = 202) as men. As for the states of residence of the sample, most of the Spanish participants, 71.9% (*n* = 358) lived in the Valencian Community, and 38.5% (*n* = 146) of Brazilians lived in Rio Grande do Sul. In addition 21.8% (*n* = 78) of Spaniards and 67.5% (*n* = 175) of Brazilians were working at home or in a different work environment than usual at the time of the data collection.

### 3.1. Content Construction and Validity Stage

The most relevant theoretical categories related to the phenomenon under study were analysed. From the literature search, an essential category for the study was outlined: RB and mental health in the digital age. A list of 40 items was obtained. Question pre-testing conducted a 21-item reduction, in order to determine the unifactorial nature of the scale. Descriptive statistics of the RB scale showed content validity and internal structure of the instrument. According to the judges’ assessment from one to five, the following means were obtained: linguistic clarity M_ean_ = 4.0, relevance M_ean_ = 5.0, relevance M_ean_ = 5.0, and classification M_ean_ = 5.0. The Finn coefficient for each aspect was above 0.79. The overall Finn coefficient score was 0.91, indicating that the judges agreed and considered the items to be consistent with the theoretical and contextual aspects. The suggestions that were made by the judges were considered, and the necessary modifications indicated by them were carried out.

### 3.2. Internal Structure

Table 1 depicts the descriptive data regarding the responses from the proposed tool, the factor loadings of the items, and the reliability measures for each item score (in both Brazil and in Spain). The items showed an adequate factor loading (i.e., ≥0.32), except for items 6 and 7, which were discarded. Results from the CFA can be described as follows: χ^2^ = 575.73, df = 152, CFI = 0.99, TLI = 0.99, RMSEA = 0.06; χ^2^ = 935.74, df = 152, CFI = 0.96, TLI = 0.95, RMSEA = 0.09.

After analysing the factorial scores of the items, items six and seven were discarded. The final tool was composed of 19 items. The results indicated high factorial loads only in one factor. In Table 2, it is possible to observe that all factor loadings were statistically different from zero, and greater than 0.57. The items can be seen in Appendix A.

### 3.3. Validity Tests of the Internal Structure of the Instrument (Comparison between Groups)

In relation to the invariance analysis, a strict level was reached in the standard model. Although in the robust model the indices showed adequate values, an increase in CFI was observed at the scalar level. The model fit indices are described in Table 3.

Reliability and internal consistency tests through Cronbach’s alpha and McDonald’s omega were adequate, both above 0.70. Note that Cronbach’s alpha represents the mean intercorrelation between the items under study, indicating that the responses were consistent.

McDonald’s Omega is based on the factor loading, and indicates that the factor explains most of the variance; therefore, 0.70 +, represents 7^2^ = 49% of the variance or more. For both measures, the reliability ranges from zero to 1.0, considered as a cut-off point to determine the reliability of the factor [42,43].

Once the evidence of validity and reliability were observed, an analysis on the item difficulty levels was performed. Table 4 contains these items, ordered by the difficulty parameter, and by their infit (level of the latent trait that the item responds to) and outfit values (level of the latent trait not represented by the item). The reliability measure of the items that were estimated by the model was 0.91. Figure 1 shows the categories fit for the assessed dimension. That is, the probability of selecting a response, and the measure of the respondent’s latent trait. The presence of this variation is desirable, since it allows for better discrimination of changes in the phenomenon, accompanied by changes in the difficulty of the items. The ordering of the stimuli shows how the construct varies in relation to the location of the item in a linear continuum; this means that the items fit was examined in terms of the linear model of measurement and reproducibility of regarding stimuli and people ordering.

The numbers 0, 1, 2, 3, and 4 were employed as sequences of repeated numbers that corresponded to each category of the rating scale. The vertical axis represents the probability of a particular response selection, where the values have a probability range from 0 to 1. The horizontal axis represents the probability that a person selects each of the responses when that person’s measure is exactly the same for the measure of the item. By indicating the difficulty of the item measured in logarithmic units, 0 represents the average difficulty; negative values represent less difficulty; and positive values represent greater difficulty [41]. Table 4 shows that 15% of the cases presented the highest RB trait, with low to moderate being the most representative category. Moreover, Figure 1 shows that the categories have distinct peaks at some point along the scale. These suggest that each category becomes the most likely option to be chosen at some point.

**Figure 2:**
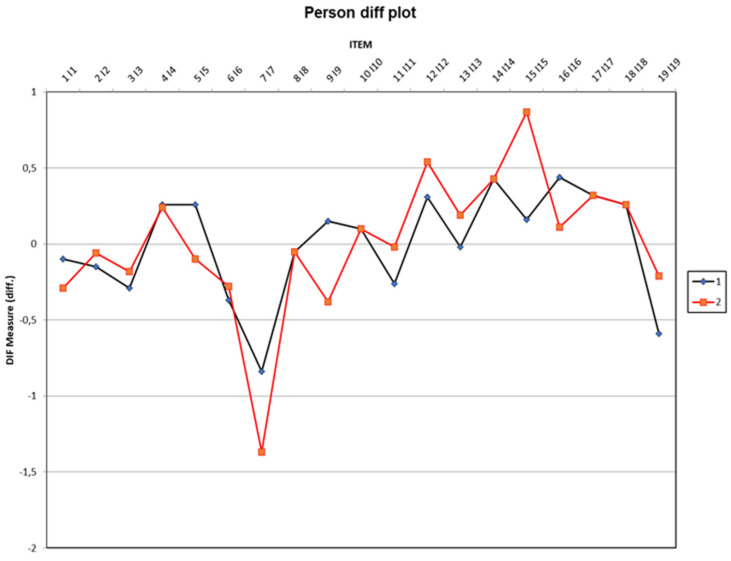
Person diff plot (differential functioning of the items by country map of items and people). Brazil = 1 [black], Spain = 2 [red]. Dots represent items’ locations (i.e. 50 percent of endorsement due to participants’ ability level) for each group. Differences lower than 0.5 are not problematic for measurement equivalence between groups.

The map of items and participants under study (Figure 2) shows the differential functioning of the items (DIF), depending on the country of the participants (Brazil = 1 [black], Spain = 2 [red]). Taking into account the country of the participants, the data revealed significant differences in item 7: “When there is an urgent problem or deadline at work I tend to use weekend time to continue working via my mobile device, tablet or computer”, the mean DIF for the Brazilian sample was −1.37 logits, while for the Spanish the value was −0.84; and for item 15: “When I have tasks to do, I am wary of posting photos of myself having fun on social net-works, even if they are while I am taking advantage of my free time”, where the mean DIF for Brazil was 0.87 logits, and for Spain it was 0.16. Even so, the items were canceled because number 7 was much easier, while number 15 was more difficult for the Spanish sample. That is, the same group had a higher probability of endorsing one item, and a lower probability of endorsing another item.

## 4. Discussion 

This study aimed to develop a tool to measure RB and its psychometric properties for different contexts, such as the Spanish and Brazilian contexts. Reliable instruments with consistent validity evidence allowed us to better understand the phenomenon of RB, and how auxiliary technology at work can influence an increase in RB. Clark and Watson [44] stated that the progress of psychological science critically depends on measurement validity. Thus, this analysis allowed us to observe that, although there were small variations in the way in which the samples of the two countries responded to the items, it was possible to verify that the reliability of the instrument did not vary in both contexts.

The theoretical basis of the instrument regarding the work-family border theory [15] and border/boundary theory [3] support the importance of the instrument and its foundation, in terms of content. This was also corroborated by the content analysis carried out by the specialist judges.

While the instrument has been shown to be adequate for evaluating RB, it is important to look at some of the items carefully. Descriptive data from the proposed scale showed that item number seven had the highest mean in comparison with all items. This is theoretically explained, as it is the item with a higher correlation with the factor because the interaction of availability demands (e.g., being contacted outside official work hours) are independent of formal norms, leading to permanent activation [45]. This may affect the management of boundaries between work and family. That is, environmental working conditions (e.g., policies, practices, deadlines) are associated with the control of limits, and have an impact on workers’ recovery experiences (control of leisure time), as well as on exhaustion [46]. Differences across countries regarding moments for leisure and work may respond to working conditions.

Item number 14 showed a negative impact on personal life, confirmed by 67.5% of the Spanish, and by 57.9% of the Brazilian sample. The empirical literature shows that in organisational cultures that expect more work outside normal hours, the beneficial effects of time control, welfare levels, and job satisfaction tend to be reduced [5]. Thus, jobs with more authoritarianism, excessive work pressures, and control of the schedule, plus RB, show a possible variability in workers’ experiences in regards to the link between technology and work-life integration [47,48].

The presence of a factor that explains RB between work and family life contributes to the understanding of this phenomenon, as conceptual uniqueness, and facilitates the relationship with the increase or decrease in the well-being of the individual. In this way, the multigroup CFA and Rasch analysis indicated similar results, which supports adequate and stable properties of the proposed work-life integration-RB scale.

In the same way, the differential functioning of items (DIF) allowed for corroboration of the items that did not have biases with respect to the country. This affords quality to the questionnaire, and supports the contribution to research practice as a measurement tool for professionals. It also supports the implementation of theoretical models, and allows for the comparison of samples in two countries with different characteristics [49].

Considering that 21.8% of Brazilians and 67.5% of Spanish participants were working at home or in a different work environment than usual at the time of collection, it is possible to say that the similar differential performance of items in both countries indicates that the scale can capture RB within and outside the context. The sudden adoption of teleworking during quarantine forced workers and their families to adapt to the new reality; therefore, these impacts should be measured [50,51].

Different limitations from the current research should be mentioned. First, it should be noted that the context of the pandemic can aggravate cases of RB. Recruitment was carried out during the post-pandemic COVID-19 period for the two countries, Brazil, and Spain, which may have influenced the results. It is suggested that more research should be conducted, with representative samples taken from various social contexts and studies outside the context of the pandemic. Other statistical methods, such as item response theory and network analysis, could provide a broader perspective. It is also suggested that more research be carried out in different countries, in order to conduct comparisons of samples. Moreover, the sampling procedure was incidental; thus, biases may have resulted. Nevertheless, we considered the current results of interest in this front. Resources provided to individuals that can be used to cushion the demands of work and their associated costs, foster challenges, help individuals achieve goals, stimulate personal growth, and encourage learning and development in different environments, are of interest in this front [8,52].

## 5. Conclusions

After proposing a new RB tool to assess work-life integration, and assessing its psychometric properties in different contexts, the main conclusions can be listed as follows: (i) the confirmatory factor analysis pointed to a unifactorial structure for both countries; (ii) there were no significant alterations in the model fit indices, and the Rasch rating scale model (RSM) showed adequate infit and outfit statistics, with successive response categories located in the expected order for all items.

Consistent measures are necessary for research practice, and for the early detection of behaviours that can generate symptoms such as anxiety and stress. In order to prevent, promote, and intervene with possible consequences that negatively affect physical and mental health, it is necessary to obtain reliable data from which to generate strategies for the management of limits.

## Figures and Tables

**Table 1:** Factor loadings for the new blurring tool.

Item	Half (*SD*) Overall	Total *λ*	Half (*SD*) BR	*λ* BR	Half (*SD*) SP	*λ* SP
1	2.23 (0.98)	0.63	2.41 (1.48)	0.61	2.11 (0.92)	0.62
2	2.18 (0.97)	0.74	2.46 (1.00)	0.78	1.98 (0.89)	0.66
3	2.25 (1.05)	0.82	2.53 (1.09)	0.86	2.06 (0.97)	0.74
4	1.99 (1.00)	0.75	2.21 (1.07)	0.79	1.84 (0.92)	0.68
5	2.10 (1.09)	0.56	2.23 (1.10)	0.57	2.00 (1.07)	0.55
6	2.32 (1.03)	0.76	2.61 (1.68)	0.78	2.12 (0.97)	0.72
7	2.80 (1.12)	0.68	2.86 (1.13)	0.76	2.76 (1.11)	0.65
8	2.15 (1.10)	0.75	2.42 (1.14)	0.81	1.97 (1.04)	0.67
9	2.22 (1.06)	0.75	2.29 (1.12)	0.82	2.17 (1.03)	0.73
10	2.08 (1.00)	0.8	2.34 (1.05)	0.83	1.90 (0.92)	0.76
11	2.19 (1.01)	0.82	2.51 (1.08)	0.85	1.97 (0.90)	0.76
12	1.91 (1.01)	0.85	2.21 (1.04)	0.86	1.70 (0.94)	0.81
13	2.08 (1.02)	0.89	2.39 (1.06)	0.92	1.87 (0.94)	0.84
14	1.91 (0.92)	0.74	2.15 (0.98)	0.78	1.74 (0.85)	0.66
15	1.87 (1.08)	0.62	2.31 (1.21)	0.68	1.56 (0.86)	0.46
16	2.01 (1.03)	0.72	2.15 (1.12)	0.79	1.90 (0.94)	0.66
17	1.97 (0.96)	0.8	2.21 (1.02)	0.81	1.79 (0.89)	0.76
18	2.00 (1.05)	0.61	2.23 (1.09)	0.65	1.84 (0.99)	0.53
19	2.35 (1.06)	0.71	2.72 (1.03)	0.74	2.09 (1.00)	0.63
Total country score			49.7 (14.8)		41.8 (11.4)	

BR = Brazil; SP = Spain; λ = factor loadings. Note: indices calculated after deleting items 6 and 7 which did not show an adequate factor loading.

**Table 2:** Standard and robust fit indices of the invariance analysis.

	Standard					Robust				
	X^2^	df	CFI	TLI	RMSEA	X^2^	df	CFI	TLI	RMSEA
baseline model	575.73	152	0.99	0.99	0.06	935.74	152	0.96	0.95	0.09
configuration	672.97	304	0.99	0.99	0.06	1005.7	304	0.96	0.95	0.08
metric	715.2	323	0.99	0.99	0.06	1028.3	323	0.96	0.96	0.08
scale	810.33	341	0.99	0.99	0.06	873.19	341	0.97	0.97	0.07
strict	810.33	360	0.99	0.99	0.06	906.82	360	0.97	0.97	0.07
**Cronbach’s Alpha** 0.94; **McDonald’s Omega** 0.94										

**Table 3:** Parameters for item difficulty and item fit.

Item	Infit	Outfit	Location
1	1.17	1.19	0.07
2	0.9	0.94	0.21
3	0.76	0.74	0.09
4	0.9	0.96	0.51
5	1.4	1.61	0.28
6	0.87	0.91	0.03
7	1.17	1.15	−0.77
8	0.94	0.92	0.2
9	0.92	0.9	0.1
10	0.78	0.76	0.35
11	0.79	0.74	0.16
12	0.82	0.81	0.65
13	0.67	0.63	0.37
14	0.92	0.89	0.69
15	1.25	1.49	0.65
16	0.98	0.97	0.49
17	0.77	0.77	0.61
18	1.27	1.44	0.51
19	1.03	1.21	−0.03

**Table 4:** Adjustment of the response categories of the Role Blurring Scale.

Category	Infit	Outfit	Percentage %
0	1.07	1.05	35
1	0.93	0.93	32
2	0.95	1	18
3	1.06	1.13	15

## Data Availability

Not applicable.

## Appendix A

Instrucciones de Aplicación

El objetivo de la siguiente escala es recoger información sobre cómo usted percibe la incidencia de la tecnología como auxiliar de trabajo en la transición entre las funciones de diferentes roles entre la familia y el trabajo. Lea los puntos siguientes y elija la alternativa que mejor corresponda a su experiencia siendo (1) la menor frecuencia y (4) la máxima.

1. Nunca o casi nunca, 2. Algunas veces, 3. La mayoría de las veces, 4. Siempre o casi siempre.

1. Cuando tengo dificultades para acceder a Internet, me preocupa tener problemas por no lograr responder a las exigencias del trabajo

Quando tenho dificuldades de acesso a internet, fico com receio de ter problemas por não conseguir responder às demandas do trabalho

When I have difficulty accessing the Internet, I am worried about having problems for not being able to respond to work demands

2. El uso de tecnologías digitales en el apoyo de mi trabajo dificulta los momentos de descanso

O uso de tecnologias digitais como apoio do meu trabalho dificulta os momentos de descanso

The use of digital technologies to support my work makes it difficult for me to have time to rest

3. Me siento agotada/o y no logro descansar, pues tengo que hacerme cargo de las exigencias del trabajo, incluso cuando estoy en casa

Me sinto fatigada/o e não consigo descansar, pois tenho que dar conta das demandas do trabalho, mesmo estando em casa

I feel exhausted and unable to rest because I have to take care of the demands of my work, even when I am at home

4. Utilizo parte del tiempo destinado a dormir o comer para trabajar, incluso cuando estoy en casa

Uso parte do tempo de sono ou alimentação para trabalhar, mesmo estando em casa

I use part of my sleeping or eating time to work, even when I am at home

5. Recibo correos electrónicos o mensajes de trabajo al móvil, incluso cuando estoy de vacaciones

Do meu trabalho enviam-me e-mails ou mensagens no smartphone, mesmo unable to resto account que esteja de férias

I receive e-mails or work messages on my cell phone, even when I am on vacation

6. Siento que mi móvil o mi ordenador me aproximan al trabajo, en casa durante la noche o los fines de semana

Sinto que meu celular ou computador me aproximam do trabalho, em casa à noite ou nos fins de semana

I feel that my cell phone or computer brings me closer to work, at home at night or on weekends

7. Cuando hay un problema urgente o un plazo límite en el trabajo tiendo a emplear tiempo del fin de semana para continuar trabajando a través de mi dispositivo móvil, tableta u ordenador

Quando há um problema ou um prazo urgente no trabalho, tendo a usar o tempo do final de semana para continuar trabalhando no meu celular, tablet ou computador

When there is an urgent problem or deadline at work I tend to use weekend time to continue working via my mobile device, tablet or computer

8. Siento presión con la exigencia de usar la tecnología como ayuda para resolver las tareas del trabajo

Sinto pressão com a exigência do uso da tecnologia como apoio para resolver as tarefas do trabalho

I feel pressure with the requirement to use technology as an aid to solve work tasks.

9. Me preocupo cuando no tengo acceso a internet para responder a las demandas del trabajo, aunque esté en mi tiempo libre

Fico preocupado quando não consigo acessar a internet para responder às demandas do trabalho, mesmo estando no meu tempo livre

I worry when I cannot access the internet to meet work demands, even when I am in my free time

10. Me resulta difícil disfrutar plenamente mis momentos de ocio debido a preocupaciones relacionadas al trabajo

Tenho dificuldade em aproveitar plenamente meus momentos de lazer devido às preocupações do trabalho

I find it difficult to fully enjoy my leisure time due to work-related concerns

11. Mis actividades de trabajo se mezclan con mis actividades en el hogar

As minhas atividades de trabalho se misturam com as minhas atividades de casa

I find it difficult to fully enjoy my leisure time due to work-related concerns

12. No logro diferenciar dónde terminan mis actividades laborales y dónde comienza mi vida personal

Não consigo diferenciar onde termina minhas atividades laborais e onde começa minha vida pessoal

I am unable to differentiate where my work activities end and where my personal life begins

13. La falta de límites en el uso de la tecnología me hace mezclar las actividades de trabajo y mi vida personal

A falta de limites no uso tecnologia me faz misturar as atividades de trabalho e a minha vida pessoal

Lack of limits in the use of technology makes me mix my work activities and my personal life

14. Considero que el uso de dispositivos digitales como apoyo en el trabajo (teléfono móvil y sus aplicaciones, portátil, correo electrónico) impacta negativamente mi vida personal

Acho que o uso de tecnologias como apoio ao trabalho (telefone celular e seus aplicativos, notebook, e-mail) impacta negativamente na minha vida pessoal

I consider that the use of digital devices to support my work (cell phone and its applications, laptop, email) negatively impacts my personal life

15. Cuando tengo tareas por hacer siento recelo de publicar fotos divirtiéndome en las redes sociales, aunque sean mientras aprovecho mi tiempo libre

Quando tenho tarefas para fazer fico com receio de postar fotos me divertindo nas redes sociais, mesmo que sejam aproveitando o meu tempo livre

When I have tasks to do, I am wary of posting photos of myself having fun on social networks, even if they are while I am taking advantage of my free time

16. Siento culpa cuando uso mi tiempo libre para divertirme y no para resolver las demandas del trabajo

Sinto culpa quando uso meu tempo livre para me divertir e não para resolver as demandas do trabalho

I feel guilt when I use my free time to have fun and not to solve work demands

17. Mis responsabilidades y tareas se mezclan, debido a los dispositivos digitales (teléfono móvil, ordenador portátil, correo electrónico, redes sociales y otras aplicaciones), y eso impacta mi rendimiento en el trabajo

Devido aos dispositivos digitais (telefone celular, laptop, e-mail, redes sociais e outros aplicativos) minhas responsabilidades e tarefas se misturam, e isso impacta meu desempenho no trabalho

My responsibilities and tasks get mixed up, due to digital devices (cell phone, laptop, email, social networks and other applications), and this impacts my performance at work

18. Cuando trabajo desde casa, desaparece la sensación de haber cumplido con los deberes al final del día

Quando trabalho em casa, desaparece a sensação do dever cumprido no final do dia

When I work from home, the feeling of having done my work disappears at the end of the day

19. Mezclar el trabajo y la vida personal afecta negativamente mi salud mental

Misturar trabalho e vida pessoal afetou minha saúde mental

Mixing work and personal life negatively affects my mental health
